# Adenoid cystic carcinoma of the lacrimal gland metastasising to the liver: report of a case

**DOI:** 10.1186/1477-7819-4-66

**Published:** 2006-09-20

**Authors:** Bashar A Zeidan, Mohammed Abu Hilal, Mohammed Al-Gholmy, Hanan El-Mahallawi, Neil W Pearce, John N Primrose

**Affiliations:** 1 Hepato Pancreatico Biliary Surgery Unit – Southampton General Hospital, Southampton, SO16 6YD, UK; 2 Department of Histopathology – Southampton General Hospital, Southampton, SO16 6YD, UK

## Abstract

**Background:**

Adenoid Cystic Carcinoma of the lacrimal gland is a rare tumour. Their aggressive behaviour, with a high-risk of local recurrence, and late distant spread of the tumour even after aggressive management has been reported. Metastasis to the liver is rare and when it occurs, it is usually part of widespread metastasis, and therefore surgical treatment is seldom considered.

**Case presentation:**

We report a rare case of an isolated liver metastasis from a lacrimal gland adenoid cystic carcinoma 20 years after resection of the primary tumour. The patient presented with right upper quadrant pain radiating to the back and shortness of breath of 3 months duration. No local recurrence was detected during a 15 year follow-up with computerized tomography (CT) of the head. Abdominal CT scan demonstrated a solitary liver tumour with no other primary source, and the bone scan was normal. The patient was treated with an extended right hemihepatectomy. The histology revealed a predominantly cribriform tumour with focal areas of basaloid type metastatic lacrimal gland adenoid cystic carcinoma.

**Conclusion:**

This case illustrates the unpredictable behaviour of adenoid cystic carcinoma and the need for a life long follow up for these patients after treatment. The possibility of surgical resection for liver metastasis from adenoid cystic carcinoma should always be considered.

## Background

Adenoid cystic carcinomas (ACC) of the lacrimal gland are rare malignant tumours accounting for 1.6% of all orbital tumours [1-3]. Despite their rarity they are the second most frequent epithelial neoplasms occurring in the lacrimal gland after pleomorphic adenomas [4].

They are slow growing tumours, which tend to spread to adjacent structures and occasionally metastasise via haematogenous spread to lungs, brain and bone in decreasing order of frequency [5-8].

There are few studies and reports on lacrimal gland ACC describing time interval to presentation of metastases and length of follow-up required [9,10]. The liver is considered a rare site of distant metastasis and when it is involved is usually as part of disseminated disease [7]. To our knowledge, neither solitary liver metastasis, nor metastatic liver resection from a lacrimal gland ACC has been reported.

We present a case of an isolated liver metastasis occurring 20 years after initial surgery for lacrimal gland ACC, with no evidence of loco regional, synchronous or metachronous distant metastasis.

## Case presentation

In 1985, a 51-year-old male, underwent a radical craniofacial resection for a left lacrimal gland adenoid cystic carcinoma, followed by postoperative radiotherapy. He was followed-up with annual head and orbital CT scans for 15 years with no signs of loco-regional recurrence. Five years after discharge from follow-up, he presented with a 3-month history of right upper quadrant pain, radiating to his back, associated with shortness of breath and reduced exercise tolerance.

**Figure 1:**
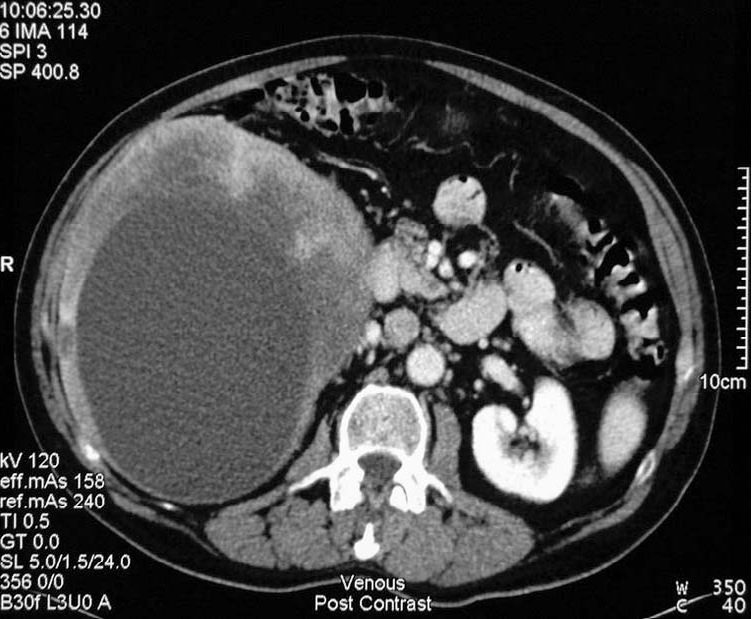
Portal venous phase image of an axial CT cut showing a large heterogeneous cystic mass within the right lobe of the liver centered on segment 5 and 6 and measuring 15 × 17 × 20 cm in maximum dimension. Further cuts show extension to segment 4A and segment 1 encroachment. No direct portal or peri-coeliac lymphadenopathy was identified.

Physical examination revealed hepatomegaly and right upper quadrant tenderness, but no stigmata of chronic liver disease. Head and neck examination revealed no sign of local recurrence. LFT, inflammatory markers, coagulation profile, CEA, CA 19-9, and AFP were all normal. An abdominal ultrasound (USS) examination and subsequent chest and abdominal CT scan showed a large 15 × 17 × 20 cm heterogeneous mass with a central area of low attenuation suggesting necrosis involving segments 4 to 8 of the liver (Figure 1), partially occluding the right portal vein. No lung or bone metastases were identified.

**Figure 2:**
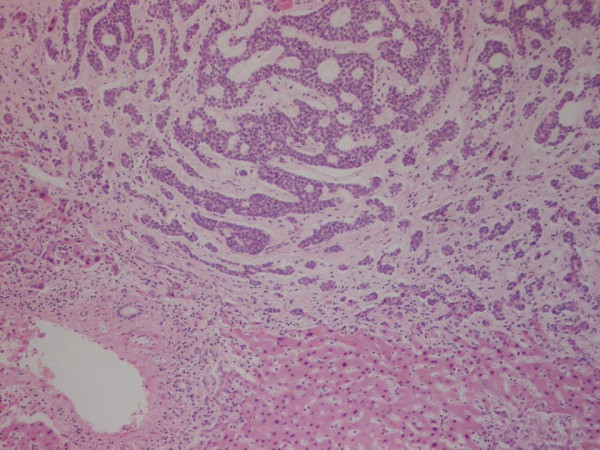
Histologically, the liver tumour comprises nests of cells with cylinderomatous microcystic spaces. These are filled with hyaline and basophilic mucoid material consistent with cribriform pattern of adenoid cystic carcinoma. Removal appears to be complete.

**Figure 3:**
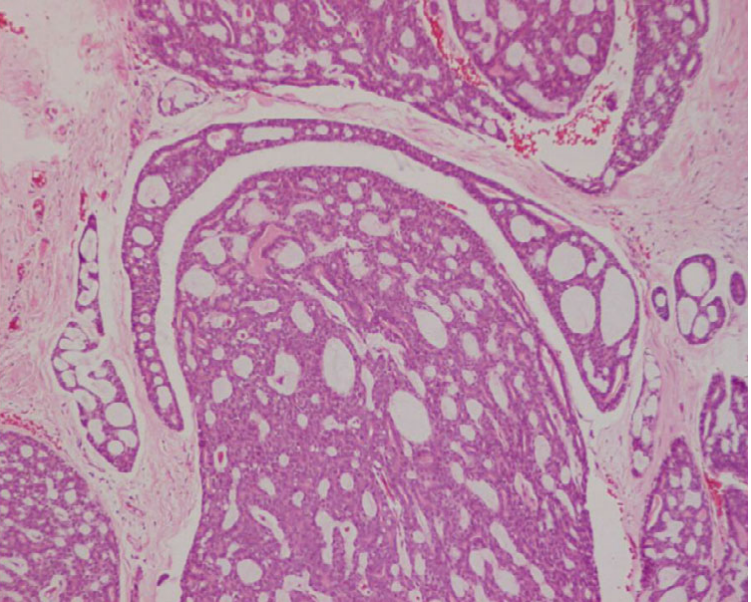
Section of the previous resected lacrimal gland tumour in 1986 was retrieved and reviewed. This shows adenoid cystic carcinoma of identical pattern to the liver tumour.

The patient was otherwise fit with no other significant past medical history. After discussion at our multidisciplinary meeting the patient was offered surgery. At operation, the tumour was found to infiltrate the right hemi diaphragm, which was locally excised *en bloc *with the extended right hemihepatectomy. The postoperative course was unremarkable and he was discharged 7 days after surgery. The histology revealed a metastatic adenoid cystic carcinoma identical to his primary lacrimal gland tumour 20 years earlier, with prominent cribriform and focal basaloid type areas (Figures 2 and 3).

## Discussion

Adenoid cystic carcinomas (ACC) of the lacrimal gland account for 1.6% of all orbital tumours [1-3]. Despite being rare, it has a well-documented dismal prognosis [11].

ACC in general has a slow biological growth and tends to have a protracted course with a poor outcome, with a reported survival rate of less than 50% at 5 years and 20% at 10 years [11-14]. Lacrimal gland ACC displays a similarly aggressive behavior with an overall survival of 20% at 10 years [15,16]. Local recurrence risk was shown to be higher than distant recurrence in 8 different studies [8,17], suggesting local recurrence as the commonest cause of disease progression and death.

In 23 years retrospective study, Ramon *et al*, reviewed the outcome of 12 patients with low grade ACC, showing that all patients had local recurrence with a mean interval of 3.25 years, and a mean survival of 5 years [18]. In the Bayler series of patients with lacrimal gland ACC, the longest survivors lived 13 and 16 years respectively after exenteration and radiotherapy [9]. In this case our patient is still alive 21 years after the primary resection.

Late presentation of distant metastases is a recognised feature of ACC with reported distant metastasis rates of 19–24%, and a mean time to presentation of 7.6 years [8]. Recurrence after 16 years has been reported [19]; with bone and lungs as the commonest sites for distant metastases [5,8,11,20].

Metastasis to the liver is rare and usually only occurs in conjunction with more widespread metastatic disease [21]. Reported rates of liver metastases in ACC are between 2–20%, with the longest reported period to presentation of only 36 months [10,21]. No isolated hepatic metastases from lacrimal gland ACC were recorded in these series.

This patient had liver metastasis as the sole presentation of secondary disease 20 years following treatment for his primary with no evidence of local recurrence. Surgical resection of liver metastasis from lacrimal gland ACC has never been reported before.

There is no proven effective chemotherapy for the treatment of metastatic lacrimal gland ACC; however some studies suggested benefit from neoadjuvant and adjuvant chemotherapy in the treatment of primary lacrimal gland ACC, in terms of improving local disease control and overall disease free survival [22].

This case highlights new aspects on ACC of the lacrimal glands, and a new evidence of the unpredictability of ACC behaviour in general.

## Conclusion

Solitary hepatic metastasis can occur extremely late after resection of lacrimal gland ACC. Life long follow-up of patients with ACC is mandatory as treatable single organ recurrence may occur decades after resection of the primary. Resection of such liver metastasis is possible and should be considered.

## Competing interests

The author(s) declare that they have no competing interests.

## Authors' contributions

**BAZ, MAH **the main operators in charge of the idea, literature search, the manuscript, article drafting and formatting.

**MAG **literature search and figures preparation.

**HEM **histological examination.

**NWP **performed surgical operation, critical revision of the article.

**JNP **critical revision and final approval of the version.

All Authors read and approved the final version of the manuscript

**Figure 4:**
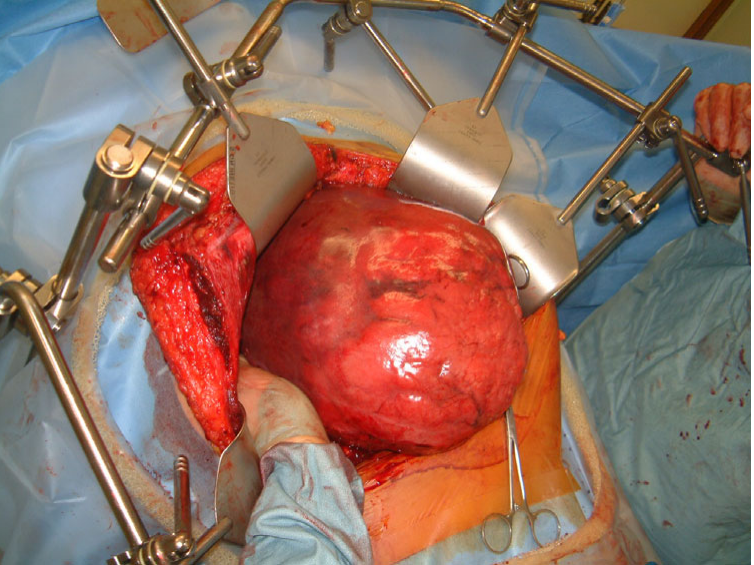
The massive liver tumor at an early stage of the challenging operation.

**Figure 5:**
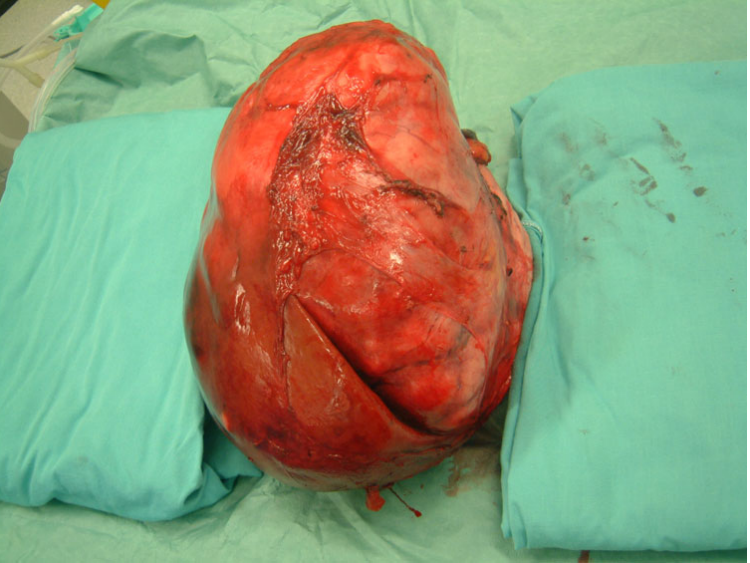
The tumour after complete resection.
